# Patient Reported Outcomes in Chronic Inflammatory Diseases: Current State, Limitations and Perspectives

**DOI:** 10.3389/fimmu.2021.614653

**Published:** 2021-03-18

**Authors:** Florian Tran, Jan Henrik Schirmer, Ilka Ratjen, Wolfgang Lieb, Philip Helliwell, Johan Burisch, Juliane Schulz, Florian Schrinner, Charlot Jaeckel, Ulf Müller-Ladner, Stefan Schreiber, Bimba F. Hoyer

**Affiliations:** ^1^ Institute of Clinical Molecular Biology, Christian-Albrechts-University Kiel, Kiel, Germany; ^2^ Department of Internal Medicine I, University Medical Center Schleswig-Holstein, Kiel, Germany; ^3^ Institute of Epidemiology and Biobank PopGen, Christian-Albrechts-University of Kiel, Kiel, Germany; ^4^ UK and Leeds Musculoskeletal Biomedical Research Unit, Leeds Teaching Hospitals NHS Trust, Leeds Institute of Rheumatic and Musculoskeletal Medicine, University of Leeds, Leeds, United Kingdom; ^5^ Gastrounit, Medical Section, Hvidovre University Hospital, Hvidovre, Denmark; ^6^ Department of Oral and Maxillofacial Surgery, University Hospital Schleswig-Holstein, Kiel, Germany; ^7^ Department of Rheumatology and Clinical Immunology, Justus-Liebig-University Giessen, Kerckhoff-Klinik GmbH, Giessen, Germany

**Keywords:** patient reported outcome (PRO), CID, IBD, precision medicine, rheumatology, mobile devices

## Abstract

Chronic inflammatory diseases (CID) are emerging disorders which do not only affect specific organs with respective clinical symptoms but can also affect various aspects of life, such as emotional distress, anxiety, fatigue and quality of life. These facets of chronic disease are often not recognized in the therapy of CID patients. Furthermore, the symptoms and patient-reported outcomes often do not correlate well with the actual inflammatory burden. The discrepancy between patient-reported symptoms and objectively assessed disease activity can indeed be instructive for the treating physician to draw an integrative picture of an individual’s disease course. This poses a challenge for the design of novel, more comprehensive disease assessments. In this mini-review, we report on the currently available patient-reported outcomes, the unmet needs in the field of chronic inflammatory diseases and the challenges of addressing these.

## Introduction - Limitations of the Current Disease Activity Measures

Chronic inflammatory diseases (CID) such as inflammatory bowel disease (IBD), rheumatoid arthritis (RA) and psoriasis are chronic inflammatory disorders at various interfaces of the human body, which, however, do not only lead to organ-specific manifestations, but indeed show strong overlaps of sites of inflammation, including the corresponding clinical symptoms. For all specific diseases, disease activity scores have been developed (e.g., SLEDAI for systemic lupus erythematodes (SLE), PASI for psoriasis). Each of these indices aim to capture organic inflammatory burden but in a relevant proportion of patients they do not reliably reflect disease activity quantified by imaging methods or invasive diagnostics, such as endoscopy in IBD. In rheumatoid arthritis (RA), even in clinical remission or low disease activity (as defined by DAS28) about 1/3 of patients is reported to have ongoing synovitis on histological and molecular level (1). The fact, that most of these indices are complex composite scores, including laboratory values, patient’s self-assessment, symptoms, and objective measures (such as the CDAI for Crohn’s disease) emphasizes the difficulties in thoroughly capturing the complexity of disease manifestation. In particular, subclinical inflammatory burden in beginning flares or insufficient disease control is hardly detectable (or distinguishable from unspecific symptoms with non-inflammatory etiology), raising the need for adequate methods for screening of these seemingly hidden disease processes. Thus, also the diagnosis of CID can be challenging if the symptoms are overseen as exemplified by the underdiagnosis of psoriasis-arthritis (PsA) by about 15% in patients with psoriasis (2), even though this population of risk (with a lifetime incidence of 6-42% of developing PsA) is easy to identify since skin manifestations precede joint disease in most of the cases.

As CID are incurable diseases and typically have an onset in young or mid-aged adults, the patients and providers need to find individual therapeutic and monitoring strategies for this problem for many decades. Unresolved chronic inflammation leads to chronic destruction of affected tissues, such as synovial tissue, cartilage and bone in RA. The current disease activity scores elude to describe or quantify the grade of irreversible tissue destruction, which accumulates over time if the inflammation is not properly controlled and accounts for long-term impairments of physical and social function. The Patient’s and Physician’s Global Assessment (PaGA/PhGA) can give a hint toward possible impairments with a higher sensitivity (3) and are thus integrated in a few scores (such as the CDAI for RA, Mayo score for ulcerative colitis), but still do not capture all possible symptoms (like psychological co-morbidities) or reflect the specific problems in the necessary granularity.

Beyond the somatic disease activity, CID also affect various aspects of life, e.g. quality of life, fatigue or social functioning and should thus be taken into account in treatment decisions by physicians (4). Of note, significant patient-physician discordance of disease activity is a frequently reported phenomenon (5, 6). This discrepancy may significantly reduce the likelihood of reaching remission in composite-scores used for measurement of disease activity, which complicates the application of treat-to-target approaches (7). Major non-inflammatory factors contributing to such discordance and, thus, complicating the interpretation of disease activity measures and reducing the likelihood of remission defined by composite scores are co-morbidities like fatigue, chronified pain-syndromes, anxiety and depression (7–9).

Therefore, in addition to objective measures (such as laboratory results) as well as the PhGA and PaGA, objectified and specified self-evaluations of disease, co-morbidities and social impairments are important to improve medical care.

## PROs: How Can the Patient’s View Help Us in Assessing Their Diseases?

Addressing this lack of structured assessment, patient-reported outcomes (PROs) are standardized and validated instruments that generate numerical data representing the patients’ perception and view on the burden of a disease and its treatment (e.g., symptoms, disease course, treatment effects) (10–13). PROs have been developed in research settings but are increasingly used in clinical practice and considered an essential part of comprehensive patient assessment. Additionally, shared decision making is an important part of modern therapeutic strategies (14, 15).

Longitudinal use of PROs may help tracking the patient’s perspective (e.g. regarding quality of life, disease activity, functional capacity, psychological health) over the course of the disease or in response to treatment modifications (10, 13). In shared decision making, PROs are one pillar of therapy guidance.

Conceptually, PRO instruments can be categorized into generic or disease-specific measures (10). Generic measures do not target a specific disease type but can be applied across different diseases and thus, allow cross-disease comparisons. Generic PROs assess, for example, overall quality of life (established and often used instruments are e.g., EuroQoL, SF-12 and SF-36) and focus on general aspects like self-care, mobility, and physical and mental function (10). Classically, PROs can be subdivided into different domains addressing different areas of life and disease symptoms i.e. fatigue, pain, depressive symptoms, movement disabilities, which are probably the biggest domains that need to be covered.

However, and different to classical objective measures of disease activity, PROs might be influenced by many other factors, as e.g., by other co-existing diseases, psychological disorders that are not related to the disease of interest, social or financial problems (16).

Disease-specific PRO measures are constructed for a specific patient population, a specific disease, functions or symptoms (10). A number of disease-specific PROs have been developed for different chronic inflammatory disease conditions, including IBD (17) and rheumatic disease conditions (18). Most of the questions in these disease-specific PROs target a respective organ system and related symptoms. In IBD, for instance, bowel movements, bloody stool and abdominal pain are obvious questions, and PROs for rheumatoid arthritis center around functional capacity, and pain.

## PROs in Rheumatology

In rheumatology, international consortia have been formed to foster the development of PROs. OMERACT (“Outcome Measures in Rheumatology”) is such an initiative (19) which recommends measures that meet certain predefined criteria (e.g. truth, discrimination and feasibility) (19). Some commonly used PROs in rheumatology are described below.

Many PROs contain one question related to the overall disease activity or the overall health, which can be rated by the patient on a Visual Analogue Scale (as a PaGA) (20). PaGA correlates moderately with more objective measures of disease activity, but is also influenced by non-rheumatic factors, such as education or the cultural background of a patient (5, 20). Because PaGA mostly focus on the disease activity in the form of symptoms and pain at the main organ side (e.g. the joints), they rarely sufficiently assess the systemic disease process which includes systemic inflammation driving somatic (e.g. vascular and metabolic disease) as well as psychological impairments. PaGA is incorporated in classical disease scores, such as Disease Activity Score with 28-joint count (DAS28) or Simplified Disease Activity Index (SDAI), both used in rheumatoid arthritis, and is, therefore, already part of a more comprehensive assessment approach (21).

High rates of anxiety and depression (about 10 to 40%) have been reported in RA and PsA (9, 22) using standardized screening instruments such as Patient Health Questionnaire (PHQ)-9 or Hospital Anxiety and Depression Scale (HADS) which have overall well diagnostic performance in rheumatic joint diseases (23). Many patients in remission as determined by DAS28 have persisting pain, pointing on one hand toward the pathophysiology of chronic joint pain in rheumatic diseases and on the other hand toward the insufficiency of end-point definitions without PROs (24). Classical PROs used in rheumatology are scores such as HAQ-DI, which focusses on activities of daily life. Other PROs used in rheumatoid arthritis (RA) are the Routine Assessment of Patient Index Data-3 (RAPID-3; covering pain, PaGA and functional impairment) and the Rheumatoid Arthritis Impact of Disease (RAID) instrument (covering pain, functional disability, fatigue, emotional well-being, sleep, coping and physical well-being) (25, 26).

The Psoriatic Arthritis Impact of Disease (PsAID) questionnaire is an instrument to measure impact of the disease (PsA) on different domains and dimensions of the patient’s health (27), including pain, fatigue, skin problems, social participation, and work and/or leisure activities (27). Further questionnaires, like the Toronto Psoriatic Arthritis Screening (ToPAS) tool (28), the Psoriasis Epidemiology Screening Tool (PEST) (29), the Psoriatic Arthritis Screening and Evaluation (PASE) (30) and the Early Psoriatic Arthritis Screening Questionnaire (EARP) (31), have been established to target the need for early detection/screening of disease processes, and their relevance have been independently validated (2, 32). PROs have been incorporated in recent therapy goal definitions and activity scores, exemplified by the Minimal Disease Activity (MDA, containing the HAQ) (33) and the PASDAS (including SF-36 questionnaire) (34).

## PROs in Inflammatory Bowel Disease

Similar to other chronic inflammatory disease conditions, IBD is characterized by relevant perception gaps between providers and patients, both for intestinal symptoms and social or functional impact of disease (35–37). The available most comprehensive measures for disease activity are the Crohn’s Disease Activity Index (CDAI) for Crohn’s disease and the Mayo score for ulcerative colitis. The CDAI, for instance, is a complex composite score but does not reflect impact of disease in the patient’s daily life (38). The patient reported 2-item (PRO2) and 3-item (PRO3) are sub-scores of the CDAI, that cover stool frequency, the presence of abdominal pain and include the patient’s general well-being (PRO3) (39) and are currently increasingly used in clinical trials. However, many available PROs might correlate well with other composite disease scores (e.g. PRO2/3 correlates well with CDAI), but do not with objective disease activity markers, such as endoscopic scores or stool biomarkers of inflammation (40–42). Therefore, an important goal is to develop PROs that correlate better and more consistently with endoscopy-defined disease activity. However, even improved PROs will not completely bridge the discrepancies of symptoms and endoscopic disease activity and thus need to be regarded as important cornerstones but not the exclusive therapy guidance parameters.

In another approach to create a more comprehensive PRO which covers both perceived disease activity and classical patient-reported functionality, a simple, rapid tool to measure disease control from the patient’s perspective was developed and validated in 2013 - the IBD-control questionnaire (43). This questionnaire comprises 13 items with the four core domains physical, social, and emotional functioning, and treatment as well as a VAS (43). Other disease-specific PROs to measures e.g. disease-specific quality of life (IBDQ) (44), fatigue (IBD-F) (45) and disability (IBD disability index) (46, 47) in patients with IBD are also available. The IBDQ considers intestinal symptoms, systemic symptoms, social aspects, and emotional aspects (44) and the IBD disability index covers body function, body structures, activities and participation, and environmental factors (46, 47). Also a range of generic PROs are commonly applied in IBD patients including instruments that measure depression and anxiety (BDI, HADS, PHQ-9) (48–53), and sleep quality (PSQI) (54, 55). These PROs have also been acknowledged as useful measures, complementary to and correlating with the CDAI or Mayo score, to produce a comprehensive disease assessment in clinical trial and real life settings (56, 57). In IBD patients, major depression is present in ~9% and major anxiety in ~18% (52). The PHQ-9 had the highest sensitivity (95%) in detecting depression and suicide ideation in a validation study among other available PROs and thus can be used as a good screening tool for depression (58), as these co-manifestations of IBD are definitely undertreated (59).

In different European countries, such as Denmark, the Netherlands and the UK, as well as in the US, e-Health tools for the monitoring of IBD have been developed, with some of them being directly linked to the health care system (60). With the use of such e-Health applications, patients can receive treatment recommendations online and the treating physician can decide – upon review of the results of the PRO that has been completed online – whether it is required to see the patient in person in the clinic. On a parallel note, a doctor’s appointment might also be unnecessary if the results of the online PRO indicate that the patient’s disease is currently inactive.

## PROs and Comorbidities

Besides the direct disease-related inflammatory burden and symptoms, other co-morbidities (either prognostically complicating disorders or diseases as consequences of long-lasting CID or independent co-morbidities) need to be more involved into the patient’s assessment as they are associated with poorer patient-reported functional status in CID. In multimorbid patients with RA the proportion of care by rheumatology specialists is reduced (61), and thus the incorporation of the treatment of several co-morbidities is increasingly reflected in multidisciplinary treatment recommendations (62). The age-adjusted Charlson Comorbidity Index (CCI(A)) is a possible tool to assess co-morbidities (63) and has been used in oncology (64) and COVID-19 (65, 66) to predict long-term outcomes. The HAQ/HAQ-DI includes physical impairments which is an established link between perception of pain, cardiovascular and mental health (67, 68), delivering a more comprehensive picture of the individual’s everyday life, while distinguishing disability due to disease activity from co-morbidity can be difficult in CID. Thus, better tools to assess the individual role of co-morbidities need to be developed.

The association of the organic comorbid “collateral damages” of chronic inflammation and neuropsychiatric dysfunctions can be mechanistically linked in an immunological manner (69). Inflammatory cytokines can lead to persistent changes in CNS immunity, subsequently facilitating alterations in CNS function and thus i.e. skew emotional states toward depression (70). In parallel, chronic systemic inflammation leads to metabolic changes favoring accelerated atherosclerosis (71, 72) and dyslipidemia (73).

## Are PROs Commonly Used in Clinical Practice?

PROs are important tools to monitor and document the patient’s health state in clinical trial settings to compare outcomes between treatment groups, without information on individual patient’s results. In clinical practice, PROs could be used as accurate and quantitative measures of the individual patient’s needs, providing an extra layer of information besides the clinical assessment to guide the long-term therapy (74). Despite the potential of PROs to improve healthcare in CID and to foster shared decision-making, they have not been broadly implemented in the clinical routine. To overcome this, pioneering efforts promoted the increasing use of PROs in the clinical routine in certain regions, such as Denmark, by using eHealth applications (60). However, some PRO measures are relatively comprehensive (covering multiple different domains) and require a significant time effort by the patient to fill them out. Therefore, shorter but reliable tools should be developed to increase the response rate and to decrease the time and effort required to complete them (13). Based on this approach, validated short forms of several questionnaires have been developed, e.g. the short IBDQ (SIBDQ) and PROMIS short forms with 5-10 items, to increase the feasibility of multiple assessments in longitudinal trials and clinical practice. The main limitations, however, are shifts in the response pattern to PROs, which might develop over time in an individual patient due to conditioning to the questionnaires and coping with own symptoms (response-shift bias) (75).

The implementation of web-based assessments like electronic questionnaires represents another way to further promote the use of PROs and to save time and resources (10, 13). Simple compound scores could be used more often and could thus play a role as part of online tracking tools for patients. By monitoring their disease activity online with a simple scoring system, added up by increasingly available point-of-care-tests (POCT), such as fecal calprotectin, can help to evaluate disease activity in a setting like the current COVID-19 pandemic. Furthermore, information from PROs can be used as a trigger to initiate further examinations, e.g. additional laboratory analyses. For example, fatigue correlates with inflammatory activity and iron deficiency in IBD patients (76). Thus, if IBD patients report fatigue, this might guide the treating physician toward further iron tests or more comprehensive assessment of disease activity.

The structured collection of longitudinal and cross-sectional data might also contribute to identifying (novel) PROs for disease prediction. Patient-reported scores have revealed to be predictive of flares of specific diseases [e.g. multiple sclerosis (77)] and, indeed, flare specific questionnaires have been developed for some diseases (78). The most important point, however, is that PROs allow us, to some extent, to identify and address the disparity between the physician’s global assessment that is far more attached to objective measures of inflammation and the patient’s perception of disease activity. In a setting, where shared decision making is the norm, this will help us to set common ground and to define common goals beyond the pure clinical definition of remission.

## What Are the Future Needs for PROs?

Defining multidimensional measures for disease activity and co-morbidities based on PROs, physician assessment, imaging studies and molecular markers remains a challenge for the future management of chronic inflammatory diseases (79). The optimal PRO needs to either capture the inflammatory burden (even if subclinical), disease-related symptoms/co-morbidities or challenging disabilities in everyday life or identify patients at risk for disease progress. Examples of such evolving PROs are the IBD-Control and the RAID instrument.

More systematically use of PROs might be promoted by the technical advances and digitalization efforts in healthcare and thus increase the test populations for better validation of tests (80). Widespread use of waiting room devices, patient “disease activity apps” and further development and use of disease-specific POCT (“inflammometers”) could improve CID healthcare.

The further development of wearable devices, which can track vital signs, motion, stress and sleeping behavior in a real-time fashion give rise to the question, whether this considerable amount of patient data can be integrated in “Next Generation PROs”. These wearables are subjects of ongoing trials in patients with neurodegenerative disorders (81). A particular example is the assessment of fatigue, which correlates well with disease activity. In-depth assessment *via* questionnaires could be related to different motion parameters to identify potential device-derived disease activity measures such as reduced daily exercise, and first trials already hint toward the benefits of device-driven therapy guidance (82).

Combining these patient-centered measures with provider’s assessments (including laboratory measures, imaging methods) to an integrative disease activity profile might be the key for precision medicine in CID care.

## Conclusions

PROs are important for clinical management and research, as they represent a cornerstone of more personalized approaches in medicine. However, depending on the specific disease entity, the available PROs only partially reflect actual disease activity as assessed by more objective criteria like endoscopic scores. The development and usage of PROs capturing disease activity more precise for individual therapy guidance is crucial and thus they need to be implemented more widely in clinical routine.

## Author Contributions

FT, IR, WL and BH conceptualized and drafted the initial manuscript. All authors reviewed and revised the manuscript. All authors contributed to the article and approved the submitted version.

## Funding

This research was supported by the DFG ExC 2167 Precision Medicine in Chronic Inflammation, and the EU Innovative Medicine Initiative 2 Joint Undertaking (“3TR”, grant agreement no. 831434; “ImmUniverse”, grant agreement no. 853995). We thank all contributors of the discussion on this within the International Symposium of the ExC. We thank Simon Imm for critical reading of the manuscript.

## Conflict of Interest

The authors declare that the research was conducted in the absence of any commercial or financial relationships that could be construed as a potential conflict of interest.
